# Prevalence and epidemiology of *Salmonella* enterica serovar Gallinarum from poultry in some parts of Haryana, India

**DOI:** 10.14202/vetworld.2015.1300-1304

**Published:** 2015-11-14

**Authors:** Devan Arora, Suresh Kumar, Naresh Jindal, Gulshan Narang, P. K. Kapoor, N. K. Mahajan

**Affiliations:** 1 Department of Veterinary Public Health & Epidemiology, College of Veterinary Sciences, Lala Lajpat Rai University of Veterinary and Animal Sciences, Hisar- 125 004, Haryana, India; 2 Department of Veterinary Public Health & Epidemiology, Disease Free Small Animal House, Lala Lajpat Rai University of Veterinary and Animal Sciences, Hisar, Haryana- 125004, India

**Keywords:** antibiogram, *salmonella* gallinarum, serotyping

## Abstract

**Aim:**

The present study was investigated to ascertain the epidemiological status of fowl typhoid (FT) in broilers in some parts of Haryana during January 2011 to December 2013.

**Materials and Methods:**

To elucidate the epidemiological status of FT in broiler chickens for the 3 years (2011-2013) and to study the prevalence of various *Salmonella* serovars in poultry on the basis of culture characteristics, biochemical features, serotyping, and their antibiogram profile from some parts of Haryana (India).

**Results:**

A total of 309 outbreaks of FT were recorded in chickens during this period. Overall percent morbidity, mortality, case-fatality rate (CFR) in broiler chicks due to FT during this period was 9.45, 6.77, and 71.55. The yearly observations were divided into quarters A (January-March), B (April-June), C (July-September) and D (October-December). Maximum number of outbreaks - 106 (34.3%) was recorded in quarter D followed by quarters B - 84 (27.3%), C - 64 (20.7%), and A - 55 (17.7%). *Salmonella* isolates (253) were recovered from disease outbreaks in broilers from different parts of Haryana. Typical morphology and colony characters on MacConkeys Lactose Agar and Brilliant Green agar, biochemical reactions, serotyping along with antibiogram profiles were able to group these isolates into 3 groups namely *Salmonella* Gallinarum (183), *Salmonella* Enteritidis (41) and *Salmonella* Typhimurium (29). The antibiogram pattern of 183 isolates of *S.* Gallinarum revealed that most of the isolates were sensitive to gentamicin (76%) followed by amikacin (72%), kanamycin (71%).

**Conclusion:**

FT is prevalent in commercial broiler flocks in different parts of Haryana and is responsible for considerably high morbidity and mortality in affected flocks. Isolation of *S.* Gallinarum (9, 12:183) from FT cases suggest it to be the primary pathogen, however, isolation of *S.* Typhimurium and *S.* Enteritidis from these cases is a major concern. The detection of *S.* Enteritidis and *S.* Typhimurium from FT cases assumes significance from public health point of view.

## Introduction

*Salmonella* enterica serovar Gallinarum (SG), the causative agent of fowl typhoid (FT) is an acute septicemic disease of chickens and other galliforme birds [1]. The epidemiology of FT and Pullorum disease caused by *S*. Gallinarum and *Salmonella* Pullorum, respectively in poultry are known to be closely associated with infected poultry eggs, particularly with regard to its transmission from one generation to another. These are the leading causes of morbidity and mortality in commercial poultry and are responsible for significant economic losses to the poultry farmers [2]. Although, there are 2541 known serovars of *Salmonella* but in India, *Salmonella* Typhimurium and *Salmonella Enteritidis* are the two most common serotypes identified in reported cases of salmonellosis from different sources [3].

Over the years, the incidence of human infection and food poisoning by *Salmonella* has increased dramatically in Europe, USA and other parts of the world. Poultry and poultry products are the major source of infection [4].

*S*. Gallinarum infection in India became prominent when it was recorded as the commonest *Salmonella* of avian origin at the National *Salmonella* Centre at Indian Veterinary Research Institute, Izatnagar. This organism has been isolated from FT affected cases from almost all the states of India [5-7].

95 isolates of *S*. Enterica belonging to *S*. Gallinarum, *S*. Enteritidis, *S*. Typhimurium, *S*. Bareilly and *S*. Paratyphi B were reported with an overall prevalence rate of 14.40% from the North eastern region of India [8]. The prevalence of salmonellosis was studied in Karnataka, Maharashtra and Tamil Nadu and the most predominant serotype was *S*. Gallinarum in 69.6% followed by *S*. Enteritidis (21.7%) [5].

Realizing its wide prevalence, the present investigation was undertaken to elucidate the epidemiological status of FT in broiler chickens for the 3 years (2011-2013) and to study the prevalence of various *Salmonella* serovars in poultry on the basis of culture characteristics, biochemical features, serotyping and their antibiogram profile from some parts of Haryana (India).

## Materials and methods

### Ethical approval

The study was conducted after prior permission and approval from Institutional Animal Ethics Committee (IAEC), Lala Lajpat Rai University of Veterinary and Animal Sciences (LUVAS), Hisar.

### Study area

The study was conducted mainly in six western and central region of Haryana, i.e., Hisar and adjoining districts (Sirsa, Fatehabad, Rohtak, Bhiwani and Jind).

### Recording of FT outbreaks

Epidemiological data related to FT in poultry during the period 2011-2013 were obtained from the Disease Investigation Laboratory, Department of Veterinary Public Health and Epidemiology, College of Veterinary Sciences, Lala Lajpat Rai University of Veterinary and Animal Sciences, Hisar. Variables taken into consideration for epidemiological studies were morbidity, mortality, case-fatality and a number of outbreaks. Quarter-wise and year-wise incidence in relation to the epidemiological indices of FT was calculated. The history of each flock was recorded. Gross pathological changes were also recorded.

### Isolation of *Salmonella* strains

Isolation of *Salmonella* was attempted from commercial broiler chickens flocks (309) suspected to be suffering from FT. The disease was presumptively diagnosed as FT on the basis of clinical signs such as sudden death, huddling, diarrhea, dullness, ruffled feathers, and gross pathological changes such as necrotic foci on the liver, mottled spleen, and enteritis.

Briefly, samples of heart blood, liver and bile were collected aseptically and impression smears were streaked on MacConkeys Lactose Agar (MLA, Hi-media) and brilliant green agar (BGA, Hi-media) plates and kept at 37°C for 24 h. After incubation, bacterial colony from each plate was subjected to Gram’s staining. Organisms giving smooth, pinpoint, pale transparent colonies (non-lactose fermenter) on MLA were further streaked on BGA (Hi-media) plates and after 24 h of incubation showed typical small, smooth, dew drop like colonies with a pink background on BGA. Culture characteristics on MLA and BGA were used for initial identification of *Salmonella* [9]. These colonies were further subjected to biochemical tests as described by Mac-Faddin [10]. From each flock, only one colony was picked up for further testing.

### Identification of *Salmonella* isolates and serotyping

Growth characteristics, morphology and motility characteristics of the *Salmonella* isolates were studied. Different biochemical tests, such as indole, methyl red, citrate, Voges–Proskauer, nitrate reduction, and carbohydrate fermentation tests, were carried out for the characterization of the organism. Carbohydrate fermentation tests included fermentation of glucose, lactose, arabinose, mannitol, and dulcitol [11]. Based on biochemical characterization, the isolates were confirmed as *Salmonella* and were maintained in the maintenance medium at 4°C for further study. Serotyping of the isolates was got done from the National *Escherichia* and *Salmonella* Centre, Kasauli, Solan (Himachal Pradesh), India.

### *In-vitro* antimicrobial sensitivity

*In-vitro* susceptibility of 183 isolates of *S*. Gallinarum organisms (based on serotyping results) to various antimicrobial agents was determined by the disc diffusion method [12] on Mueller-Hinton agar plates (Hi-media). 16 antibiotic discs (Hi-media) of standard concentrations namely amikacin (30 mcg), ampicillin (10 mcg), ampicillin–sulbactam (10 mcg), co–trimoxazole (25 mcg), ciprofloxacin (5mcg), chloramphenicol (30 mcg), cefotaxime (30 mcg), ceftriaxone (10 mcg), enrofloxacin (5 mcg), carbenicillin (100 mcg), nalidixic acid (30 mcg), norfloxacin (10 mcg), spectinomycin (100 mcg), tetracycline (30 mcg), sulfafurazole (300 mcg), kanamycin (30 mcg), and gentamicin (10 mcg) were used. The plates were incubated at 37°C for 24 h. Results were recorded using antibiotic zone scale and interpreted as sensitive (S), and resistant (R)based on values given in zone size interpretative chart (Hi-media, India).

## Results

During 3-year period (2011-2013), a total of 309 (2.70%) flocks out of 11404 flocks brought for disease investigation, were affected with FT. The epidemiology of FT with respect to different variables like percent morbidity, mortality and CFR in broiler chicks in Haryana state during 2011-2013 have been presented in Table-1.

**Table-1:** Year wise percent morbidity, mortality, case fatality rate due to FT in broiler chicks in some parts of Haryana during 2011-2013.

Year	Total number of flocks affected	Total number of flocks affected with FT (%)	Morbidity (%)	Mortality (%)	Case fatality rate (%)
2011	4578	117 (2.55)	10.14	6.42	63.32
2012	3805	121 (3.18)	10.97	8.37	76.32
2013	3021	71 (2.35)	7.06	5.35	75.80
Total	11404	309 (2.70)	9.45	6.77	71.55

Means with different superscript in a column for a parameter differ significantly (p≤0.05), FT=Fowl typhoid

Overall percent morbidity, mortality, CFR in broiler chicks due to FT during the 3-year period was 9.45, 6.77 and 71.55, respectively. Percent morbidity due to FT was significantly higher during the years 2011 and 2012 as compared to the year 2013. Likewise, percent mortality was significantly higher in the year 2012 as compared to that in 2013. In contrast, CFR was significantly higher during the years 2012 and 2013 than that in 2011.

### Quarter-wise distribution of FT

Quarter-wise distribution of FT in relation to percent morbidity, mortality, CFR and number of outbreaks during the period from 2011 to 2013 has been shown in Table-2.

**Table-2:** Quarter wise distribution of percent morbidity, mortality, case fatality rate due to FT in broiler chicks in some parts of Haryana during 2011-2013.

Quarter	Total number of flocks affected (%)	Morbidity (%)	Mortality (%)	Case fatality rate (%)
A	55 (17.7)	10.28	6.24	60.75^b^±9.61
B	84 (27.3)	10.25	7.50	73.19^a^±4.30
C	64 (20.7)	6.87	4.84	70.37^a^±3.57
D	106 (34.3)	10.17	7.77	76.43^a^±2.14
Total	309	9.45	6.77	71.55

A=January-March, B=April-June, C=July-Sept., D=October-December, means with different superscript in a column differ significantly (p≤0.05), FT=Fowl typhoid

A maximum number of outbreaks (106) were recorded in quarter D followed by quarters B (84), C (64) and A (55). Percent morbidity and mortality in quarter C were significantly lower than quarters B and D. Though these indices were also lower in quarter C as compared to quarter A; the difference for percent mortality was not statistically significant. The CFR in quarters B, C and D was significantly higher than that in quarter A.

### Serotyping and *in vitro* antimicrobial sensitivity

Serotyping of 253 (Table-3) *Salmonella* isolates revealed that *S*. Gallinarum was the most prevalent (183 isolates; 69.62%) organism from FT affected birds followed by *S*. Enteritidis (41 isolates; 16.45%) and *S*. Typhimurium (29 isolates; 13.92%). The antigenic structure of *S*. Gallinarum was 9,12:-:- while that of *S*. Enteritidis and *S*. Typhimurium were 9,12:g,m:- and 4,12:1:1,2, respectively. The prevalence of *S*. Enteritidis and *S*. Typhimurium from FT cases assumes significance from public health point of view. The antibiogram pattern (Table-4) of the 183 isolates of *S*. Gallinarum revealed that most of the isolates were sensitive to gentamicin (76%) followed by amikacin (72%), kanamycin (71%), chloramphenicol (71%) and streptomycin (70%).

**Table-3:** Distribution of different serotypes of *Salmonella* isolated from poultry from some parts of Haryana.

Serotypes	Number isolated	Relative occurrence (%)	Antigenic structure
S. Gallinarum	183	69.62	9,12:–:–
S. Enteritidis	41	16.45	9,12:g, m:–
S. Typhimurium	29	13.92	4,12:1:1,2
Total	253	-	-

*S.* Gallinarum*=Salmonella* Gallinarum*, S. Enteritidis=Salmonella* Enteritidis*, S.* Typhimurium*=Salmonella* Typhimurium

**Table-4:** *In-vitro* antimicrobial drug sensitivity (%) pattern of *S.* Gallinarum isolated from FT cases during the year 2011-2013.

Drug	Sensitive* (%)
Gentamycin	139 (76)
Amikacin	132 (72)
Kanamycin	130 (71)
Chloramphenicol	130 (71)
Streptomycin	128 (70)
Co–Trimoxazole	126 (69)
Amoxy–Clav	126 (69)
Sulfafurazole	124 (68)
Ampicillin	122 (67)
Enrofloxacin	120 (66)
Ampicillin–Sulbactam	120 (66)
Cefoperazone	110 (60)
Norfloxacin	51 (28)
Tetracycline	38 (21)
Ciprofloxacin	37 (20)
Carbenicillin	37 (20)
Ceftriaxone	31 (17)
Cefotaxime	27 (15)
Nalidixic acid	22 (12)

Total isolates of *S.* Gallinarum tested=183, FT=Fowl typhoid, *S.* Gallinarum*=Salmonella* Gallinarum

## Discussion

Control of FT is difficult due to the endemicity of the disease [13], facultative intracellular nature of the organism, both vertical [14] and horizontal [15] modes of transmission, multiple drug resistance and presence of carrier stage. The indiscriminate and widespread use of antibiotics in the treatment of poultry diseases has lead to increase in the number of resistant *Salmonella* strains isolated [16]. Antimicrobial resistance is nowadays a global public health concern [17]. FT caused by *S*. Enterica SG is one of the most important bacterial diseases of poultry. This disease occurs more often in acute form in young chicks and mortality is encountered most frequently during first 2 weeks of age. A total of 309 outbreaks of FT were recorded in the present study with percent morbidity and mortality of 9.45 and 6.77, respectively in 3-year period. Overall CFR due to FT in these outbreaks during the 3-year period was 71.55% (Table-1). 198 outbreaks were recorded of FT in commercial broiler chicks during the period from 1987-1990 with percent mortality of 10.54%[18]. Likewise, during the period from July 1996-June 1997 recorded 39 outbreaks of FT with overall morbidity and mortality of 14.22 and 12.12%, respectively[19]. A total of 23 *Salmonella* isolates were recovered from different disease outbreaks in different geographical locations of Karnataka, Maharashtra and Tamil Nadu [5]. 227 outbreaks of FT were recorded from January 2005 to December 2008 with percent mortality and CFR ranging from 1.27 to 6.00 and 67.09 to 76.14, respectively [2].

The occurrence of FT was higher in October-December with significantly higher morbidity and mortality. Though percent morbidity was similar throughout the year (except July to September), however, percent mortality was higher in October to December months with significant difference from July to September. Many workers have [2,18] also recorded higher mortality in winter months. Though the disease was recorded throughout the year; higher occurrence and mortality in October to December months could be due to harsh weather conditions particularly in December thereby causing stress on birds and making birds more susceptible to disease.

Vertical transmission of infection from breeding hens to progeny is an important aspect of the epidemiology of *Salmonella* spp. infection within the poultry industry [20]. Being vertically transmitted disease, most FT outbreaks are recorded in young broiler chicks in this region. Extreme weather conditions and improper management may lead to higher mortality in chicks. Hence, it is important that the chicks should be procured from hatchery that is free from *Salmonella*.

The results of serotyping revealed increased the prevalence of *S*. Gallinarum than *S*. Enteritidis and *S*. Typhimurium in FT affected birds in this region (Table-3). *S*. Gallinarum, the causative agent of FT, is the most prevalent host adapted *Salmonella* strain of poultry in India [21]. *S. enterica* SG infections have been reported from time to time in many parts of the world by various workers notably from Canada[22] and from England [23].

Many workers also reported more isolations of *S*. Gallinarum than *S*. Enteritidis from poultry and *S*. Enteritidis or *S*. Typhimurium in addition to *S*. Gallinarum have been also isolated from FT affected birds from abroad [24,25]. The detection of *S*. Enteritidis and *S*. Typhimurium from FT cases assumes significance from public health point of view [26,27].

The antibiogram of 183 isolates of SG revealed that most of the isolates were sensitive to gentamicin followed by amikacin, kanamycin and chloramphenicol, the pattern was more or less in accordance with the findings of [3,28]. Above all, maximum resistance was obtained against nalidixic acid followed by carbenicillin [29]. A study also showed a high prevalence of nalidixic acid resistance among *Salmonella* isolates [30]. It is surprising to note that SG isolates were resistant to nalidixic acid whose use in poultry feed as feed additive or for treatment purposes seems to be rare in this region. Development of resistance by the organisms to other antimicrobials could be due to their indiscriminate use in feed as additives or for treatment purposes [31]. Hence, there is a need to educate farmers that the antimicrobials should be used judiciously, and indiscriminate use should be discouraged.

## Conclusions

FT is prevalent in commercial broiler flocks in different parts of Haryana and is responsible for considerably high morbidity and mortality in affected flocks. Isolation of *S*. Gallinarum (9, 12:-:-) from FT cases suggest it to be the primary pathogen, however, isolation of *S*. Typhimurium and *S*. Enteritidis from these cases is a major concern. The majority of isolates were resistant to nalidixic acid and carbencillin while most sensitive antibiotics were gentamicin, amikacin and kanamycin. Surveillance, identification and antibiotic sensitivity of the prevalent *Salmonella* serotypes in the country would help devise suitable prevention and control program for this important poultry pathogen. Since the consumption of poultry products is often associated with salmonellosis, therefore, it becomes necessary to update information about *Salmonella* resistance to antibiotics used in poultry production.

## Authors’ Contributions

DA and SK participated in the epidemiological studies. NJ and GN collected bacterial isolates. PKK, NKM carried out biochemical characterization and ABST. All authors contributed in drafting and revision of the manuscript. All authors read and approved the final manuscript.
